# Importance of 14-3-3eta, anti-CarP, and anti-Sa in the diagnosis of seronegative rheumatoid arthritis

**DOI:** 10.3906/sag-1812-137

**Published:** 2019-10-24

**Authors:** Emrah SALMAN, Salih ÇETİNER, Barış BORAL, Filiz KİBAR, Eren ERKEN, Emine Duygu ERSÖZLÜ, Suade Özlem BADAK, Reyhan BİLİCİ SALMAN, Yaşar SERTDEMİR, Alev ÇETİN DURAN, Akgün YAMAN

**Affiliations:** 1 Department of Immunology, Ankara City Hospital, Ankara Turkey; 2 Department of Immunology, Faculty of Medicine, Çukurova University, Adana Turkey; 3 Department of Immunology, Adana City Hospital, Adana Turkey; 4 Department of Medical Microbiology, Faculty of Medicine, Çukurova University, Adana Turkey; 5 Department of Rheumatology, Faculty of Medicine, Çukurova University, Adana Turkey; 6 Department of Rheumatology, Adana City Hospital, Adana, Turkey; 7 Department of Rheumatology, Faculty of Medicine, Gazi University, Ankara Turkey; 8 Department of Biostatistics, Faculty of Medicine, Çukurova University, Adana Turkey; 9 Department of Immunology, Aydın State Hospital, Aydın Turke

**Keywords:** 14-3-3eta, Anti-carP, Anti Sa, seronegative

## Abstract

**Background/aim:**

Rheumatoid arthritis (RA) is an autoimmune disease characterized by synovial inflammation. The study aimed to assess serum 14-3-3eta, anti-CarP, and anti-Sa in seronegative RA (SNRA) patients who were treatment-naïve as well as in healthy subjects. This is the first study in the literature to examine these autoantibodies together in SNRA patients.

**Materials and methods:**

Forty-five treatment-naïve SNRA patients and 45 healthy subjects were recruited. Drugs change the levels of autoantibodies; therefore, patients who took any medication had been excluded from our study. Anti-carbamylated protein, anti-Sa, and 14-3-3eta were measured by using three different ELISA kits.

**Results:**

Median serum concentration of healthy controls in 14-3-3eta was 0.02 (0.02–0.27) ng/mL. Median serum concentration of SNRA patients in 14-3-3eta was 1.00 (0.48–1.28) ng/mL. Data were analyzed with Mann–Whitney U tests; the P-value was <0.001 in 14-3-3eta. Receiver operating characteristic (ROC) curve analysis showed that 14-3-3eta in SNR compared to healthy controls had a significant (P < 0.001) area under the curve (AUC) of 0.90 (95% confidence interval, 0.83–0.96). At a cutoff of ≥0.33 ng/mL, the ROC curve yielded a sensitivity of 88.9%, a specificity of 82.2%, a positive predictive value of 83.3%, and a negative predictive value of 88.1%.

**Conclusion:**

We found that 14-3-3eta can be used as a diagnostic marker in SNRA.

## 1. Introduction

Rheumatoid arthritis (RA) is an autoimmune disease characterized by synovial inflammation which may lead to irreversible joint damage, decreased mobility, and reduced quality of life [1]. Seronegative RA (SNRA) is the diagnosis of RA without specific antibodies in the blood. If test results are negative for rheumatoid factor (RF) and cyclic citrullin peptide (aCCP) antibodies but patients nevertheless have pronounced symptoms of RA, they can be diagnosed as having SNRA [1]. Today, RA is classified according to a set of criteria defined by the American College of Rheumatology (ACR) [2]. These criteria were recently revised by the ACR and the European League Against Rheumatism (EULAR) committees [3]. According to the updated criteria, the presence of antibodies against two RA disease markers—RF and aCCP—is an important criterion for the diagnosis of RA. Recent metaanalyses indicate that one-third of RA patients are seronegative for these two markers [4,5]. Seronegativity in cases of both early and established RA remains an important limitation of these two disease markers, emphasizing the need for new complementary markers to enhance diagnostic sensitivity [6]. New markers are needed to better classify patients in different risk categories, because current markers account for only 32% of the total variance in the prediction of joint destruction [7].

The ligand activity of soluble 14-3-3eta preferentially activates cells of the innate immune system. This protein acts via signaling cascades (such as the extracellular signal-regulated kinase and p38 pathways) to upregulate proinflammatory cytokines, including interleukin 1β (IL-1β), IL-6, tumor necrosis factor (TNF alpha), and other factors involved in joint degradation such as MMP-9 and the receptor activator of nuclear factor-kB ligand (RANKL) [8]. The carbamylation of lysine residues to form homocitrulline may be a key mechanism triggering inflammatory responses. Carbamylated antigens have been reported to activate T cells and thereby assist in T-cell–mediated antibody production [9]. Recent observations have shown that vimentin causes cell death in human macrophages. This makes citrullinated vimentin and antibodies against this antigen (such as anti-Sa) promising candidates for use in the diagnosis of RA. Further research may provide new information about the potential role of citrullinated synovial antigens and antibodies in the pathophysiology of RA [10]. The study aimed to assess serum 14-3-3eta, anti-CarP, and anti-Sa in SNRA patients who were treatment-naïve and in healthy subjects.

## 2. Materials and methods

This cross-sectional study was performed between April and November 2017. Forty-five healthy volunteers and 45 SNRA patients were admitted to the internal medicine–rheumatology departments of the Çukurova University School of Medicine and Adana City Hospital. Newly diagnosed and untreated with conventional synthetic disease-modifying antirheumatic drugs (DMARDs), glucocorticoids, and biological DMARDs seronegative rheumatoid arthritis patients were included in the study. The exclusion criteria for seronegative rheumatoid arthritis were the presence of chronic infections, seropositive rheumatoid arthritis, connective tissue diseases, psoriatic arthritis, spondyloarthritis, and other systemic diseases. The exclusion criteria for healthy volunteers were the presence of chronic kidney disease, hepatic dysfunction, rheumatological diseases or chronic infections. Healthy volunteers were recruited to set the 14-3-3eta, anti-CarP, and anti-Sa antibody thresholds. 

The Declaration of Helsinki protocols were followed and approval for the study was granted by the Çukurova University Hospital Ethics Committee (Ref 2017; 64). All participants gave written informed consent. We used the 1987 ACR criteria or the 2010 ACR/EULAR criteria as diagnostic references. Serum samples were collected and spun at 4000 rpm for 4 min and then aliquoted and stored at –20 °C. Rheumatoid factor was measured by a nephelometric method in the immunology laboratory at the Çukurova University Balcalı Hospital. aCCP was measured by CCP-2 and/or CCP-3 enzyme-linked immunosorbent assay (ELISA) (Inova). Tests were carried out and results interpreted according to the manufacturer’s recommendations. Positive samples for either aCCP-2 or aCCP-3 were considered as aCCP-positive. The 14-3-3eta antibodies, anticarbamylated protein antibodies, and anti-Sa antibodies were measured with three different ELISA kits: 1) Cusabio, Wuhan, China; 2) Novateinbio, Woburn, MA, USA; and 3) Euroimmun, Lübeck, Germany. These assays employ the quantitative sandwich enzyme immunoassay technique.

Statistical analysis was performed using the SPSS 22.0 (IBM, Chicago, IL, USA) computer program. In statistical analysis, categorical variables were given as numbers and continuous variables were presented with median (interquartile range = 25th percentile to 75th percentile) for descriptive analyses. The conformity of continuous variables to normal distribution was evaluated using visual (histogram and probability graphs) and analytical methods (Kolmogorov–Smirnov/Shapiro–Wilk tests). Normality analysis revealed that all data sets were not distributed normally. The Mann–Whitney U test was used for comparison of data sets which were not normally distributed for the variables. Receiver operating characteristic (ROC) analysis was used to determine if any of these 3 immune markers may participate in seronegative rheumatoid arthritis. The sensitivity, specificity, positive predictive value, and negative predictive value of significant limit values were estimated. P < 0.05 was considered statistically significant.

## 3. Results

Forty-five patients with SNRA and 45 healthy volunteers were analyzed. Basic characteristics of the study population are given in Table 1. Groups were similar in terms of demographic parameters.

**Table 1:** Demographic data and laboratory findings of the seronegative rheumatoid arthritis (SNRA) cases and healthy controls.

	SNRA cases	Healthy controls	P-value
Number of cases	45	45	
Male/Female	36/9	36/9	
Age (years)	53.0 (46.0–60.5)	52 (43.0–59.0)	0.42

14-3-3 eta protein (ng/mL) 1.00 (0.48–1.28) 0.02 (0.02–0.27) <0.001 Anti-CarP (ng/mL) 1.23 (0.88–1.79) 1.47 (0.87–2.22) 0.27 Anti-Sa (ng/mL) 9.29 (6.12–13.69) 8.86 (6.63–14.7) 0.47

Median serum concentration of 14-3-3eta in healthy controls was 0.02 (0.02–0.27) ng/mL and 1.00 (0.48 –1.28) ng/mL in SNRA patients. There was a statistically significant difference between the SNRA and control groups in 14-3-3eta; the P value was <0.001.

When other serum autoantibodies for SNRA patients (anti-CarP and anti-Sa) were compared with those of the healthy group, there was no statistically significant difference between the groups. Median serum concentration of anti-CarP in healthy controls was 1.47 (0.87–2.22) ng/mL and 1.23 (0.88 –1.79) ng/mL in SNRA patients. Median serum concentration of anti-Sa in healthy controls was 8.86 (6.63–14.7) ng/mL and 9.29 (6.12 –13.69) ng/mL in SNRA patients. 

In this study, whether 14-3-3eta, anti-CarP, and anti-Sa values are predictive of the disease was evaluated by ROC analysis (figure). Area under the curve (AUC), cutoff, positive likelihood ratio, sensitivity, specificity, positive predictive value, and negative predictive value are presented in Table 2. ROC curve analysis showed that 14-3-3eta in SNR compared to healthy controls had a significant (P < 0.001) AUC of 0.90 (95% CI, 0.83–0.96). At a cutoff of ≥0.33 ng/mL, the ROC curve yielded a sensitivity of 88.9%, a specificity of 82.2%, a PPV of 83.3%, and an NPV of 88.1%. The mean serum level of 14-3-3eta was 1.00 (0.48 –1.28) ng/mL in SNRA patients.

**Figure:**
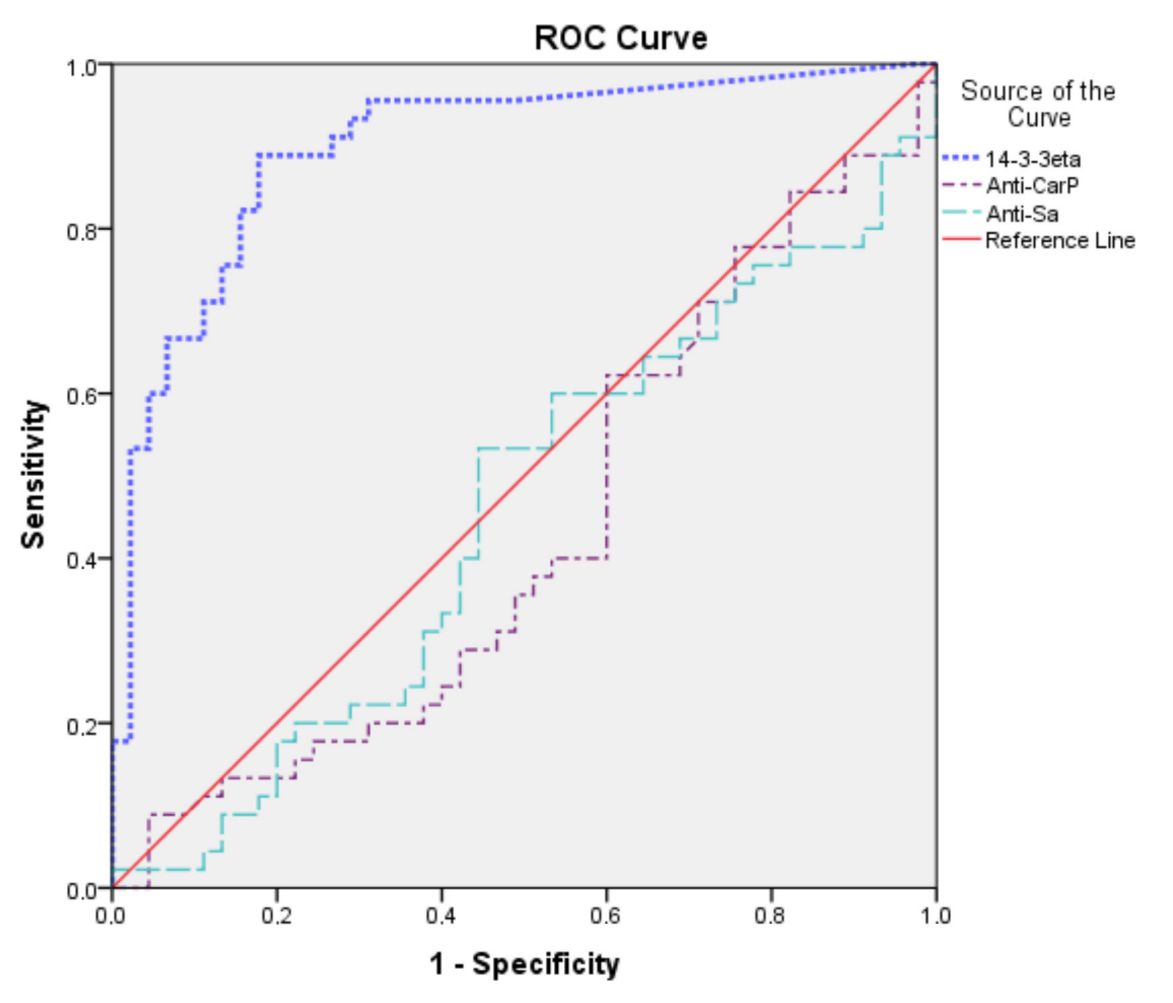
ROC-curve for 14-3-3eta as a predictor of seronegative rheumatoid arthritis.

**Table 2:** Statistical parameters of various diagnostic approaches for predicting in patients with seronegative rheumatoid arthritis.

Parameter	AUC(95% CI)	P	Cutoff	Positivelikelihood ratio	Sensitivity	Specificity	PPV	NPV
14-3-3eta	0.90 (0.83–0.96)	<0.001	≥0.33	5.0	88.9%	82.2%	83.3%	88.1%
Anti-CarP	0.43 (0.31–0.55)	0.27	≤1.09	1.03	62.2%	40.0%	54.8%	52.5%
Anti-Sa	0.45 (0.33–0.57)	0.47	≥8.22	1.13	60.0%	46.7%	52.9%	53.8%

PPV, positive predictive value; NPV, negative predictive value.

Accordingly, it was found that only 14-3-3eta measurements of the three parameters had statistically significant diagnostic power. According to ROC analysis, positive likelihood ratio, sensitivity, and specificity were found to be highest for 14-3-3eta; the cutoff was 0.33 ng/mL.

## 4. Discussion

In the present study, we found that 14-3-3eta can be used as a diagnostic marker in SNRA. New markers are needed for early diagnosis of rheumatoid arthritis as seronegativity in both early and settled RA remains a major limitation of both anticitrullinated protein antibodies and rheumatoid factor. Both aCCP and RF tests are included in the ACR/EULAR classification criteria for RA [11]. Even though the aCCP test is more specific than that for RF, studies have shown that the combined use of markers means greater sensitivity is maintained than would otherwise be the case if a single marker was used [12,13]. Despite this increased sensitivity, relatively few patients test positive for RF (28%) and aCCP (44%) in the early stage of the disease. Patients who develop erosive RA may also remain negative for both of the markers [14–16]. Therefore, new markers are needed to assist in the diagnosis of RA.

Extracellular 14-3-3eta activates key signaling cascades and induces factors associated with the pathogenesis of RA and plays a role in stimulating tumor necrosis factor alpha, metalloproteinases, and other inflammatory mediators that are important in the joint erosive process [14]. One of the advantages of 14-3-3eta as an RA marker is that it can improve identification rates of early RA. The median serum concentration of 14-3-3eta was reported to be 6.13 ng/mL in early RA patients with joint damage (n = 13) and 1.30 ng/mL in those without joint damage (n = 20) [15]. In a different study, 6 (21%) of 28 patients with SNRA were 14-3-3eta–positive. The mean serum level of 14-3-3eta in these 6 patients was 3.98 ng/mL, with a range of 0.35–12.65 ng/mL (normal <0.20 ng/mL) [17]. 

There is a limited study in this area concerning 14-3-3eta in SNRA. Naides and Marotta investigated 14-3-3eta in only 28 SRNA patients and found 21% 14-3-3eta positivity; they claimed that 14-3-3eta together with ACPA and RF can aid in the early detection of RA. In our study, 40 (88%) of the 45 patients who were seronegative for RF and ACPA were 14-3-3eta–positive. Our study included more SNRA patients than others, and we showed the best sensitivity for 14-3-3eta in the literature.

Maksymowych et al. found that adding 14-3-3eta to RF and CCP antibody testing increased diagnostic sensitivity for early RA patients [14]. ROC curve analysis comparing established RA with healthy subjects demonstrated a significant (P < 0.0001) AUC of 0.89 (95% CI, 0.85–0.9). At a cutoff of ≥0.19 ng/mL, the ROC curve yielded a sensitivity of 77.0%, a specificity of 92.6%, an LR positivity of 10.4, a PPV of 0.70, and an NPV of 0.80. When comparing established RA with all controls, the same cutoff yielded a sensitivity of 77.4% and specificity of 86.0%. 

Mohamed and colleagues found that adding 14-3-3eta to RF and CCP antibody testing increased diagnostic sensitivity for early RA patients. ROC curve analysis comparing established RA with healthy subjects demonstrated a significant (P < 0.0001) AUC of 0.999 (95% CI, 0.997–1.00). At a cutoff of ≥0.39 ng/mL, the ROC curve yielded a sensitivity of 87.7%, a specificity of 97.6%, a PPV of 0.98, and an NPV of 0.85, meaning that 14-3-3eta is more specific in early RA with high NPV. On the other hand, ACPA has a PPV 95% for the development of RA in patients with undifferentiated arthritis, although its NPV is only about 60–70% [18].

ROC curve analysis showed that 14-3-3eta in SNRA compared to healthy controls had a significant (P < 0.001) AUC of 0.90 (95% CI, 0.83–0.96). At a cutoff of ≥0.33 ng/mL, the ROC curve yielded a sensitivity of 88.9%, a specificity of 82.2%, a PPV of 83.3%, and an NPV of 88.1%.

 In the literature, there have been a few studies on the relationship between 14-3-3eta and seropositive early RA. However, there have been no studies comparing SNRA and healthy controls for 14-3-3eta, anti-CarP, and anti Sa. Patients with undistinguishable seronegative arthropathy require testing serum 14-3-3eta for early detection of RA, which will be of great advantage. 14-3-3eta is a valuable and promising marker in patients with SNRA.

There are a limited number of studies investigating autoantibodies such as anti-CarP and anti-Sa in patients with SNRA. Anti-CarP was observed in sera collected from healthy subjects many years before the development of RA in those subjects. The observation of anti-CarP in the preclinical and early stages of the disease suggests a role (as yet not fully clarified) for these antibodies in the pathogenesis of RA. Anti-CarP IgG antibodies were found to be related with a more severe radiological progression in cases of aCPP-negative RA [19]. In our study, we found that there was no statistically significant difference between the serum concentrations of anti-CarP antibodies in SNRA patients and healthy controls (P = 0.27). In another study, the presence of anti-Sa antibodies in serum was shown to possibly be useful as a complementary assay when anti-CCP antibodies are negative and RA is suspected [20]; however, in our study, we found that SNRA patients and healthy controls were not significantly different with respect to serum concentrations of anti-Sa antibodies (P = 0.47). 

Our results suggest that anti-CarP and anti-Sa antibodies cannot be used as a diagnostic marker in SNRA, but these results do not provide enough scientific data to form a conclusion. More comprehensive work is needed. A limitation of our study is the small number of seronegative patients included, which limits the statistical power.

In conclusion, 14-3-3eta, anti-CarP, and anti-Sa have been evaluated individually in the literature in early seropositive RA. However, we evaluated 14-3-3eta, anti-CarP, and anti-Sa in newly diagnosed RF and ACPA negative patients and showed the best sensitivity for 14-3-3 eta. SNRA remains poorly diagnosed in the absence of specific antibodies; 14-3-3eta could be clinically useful in patients with SNRA in the future.

## Acknowledgments

This study was supported by the Research Fund of the Çukurova University Scientific Investigation Projects Office.
